# Single-cell quantification of functional outcomes of CD16 ligation in NK cells by Fc-engineered antibodies

**DOI:** 10.1186/2051-1426-1-S1-O5

**Published:** 2013-11-07

**Authors:** Gabrielle Romain, Vladimir V Senyukov, Ivan Liadi, William Kelton, George Georgiou, Dean A Lee, Navin Varadarajan

**Affiliations:** 1 Chemical and Biomolecular Engineering, University of Houston, Houston, TX, USA; 2 MD Anderson Cancer Center, University of Texas, Houston, TX, USA; 3 Institute for Cell and Molecular Biology, University of Texas, Austin, TX, USA

## 

Humanized monoclonal antibodies (mAb) targeting tumor antigens have paved the way to complementary strategies in the treatment of cancer by harnessing the immune system towards a more specific response against tumors. Such mAb can mobilize natural killer (NK) cells function by mediating antibody-dependent cell cytotoxicity (ADCC). ADCC can be enhanced through Fc-region modification, but high affinity-Fc mAb have been associated with NK cell depletion. Here, we have quantified in vitro the improvement of NK cell-mediated ADCC brought by a humanized mAb targeting CD33 (HuM195) engineered in its Fc domain (DLE mutation). We used human NK cells in cytotoxicity assays against murine EL4 tumor cells transfected with human CD33 and pre-coated with the DLE HuM195 anti-CD33 antibody. In parallel, human K562 cells were used as targets in comparative assays to quantify kinetics of direct tumor recognition. A single-cell assay combining high throughput microscopy and immuno-printing of thousands of NK - target cell interactions allowed us to observe effector-mediated apoptosis and to measure cytokine secretion. We demonstrated that the DLE HuM195 anti-CD33 antibody increased NK cell-mediated ADCC up to 3 times compared with the non-mutated HuM195 anti-CD33 in a bulk cytotoxicity assay. In the single-cell assay, we confirmed that the DLE-engineered HuM195 potentiated the cytotoxicity of NK cells against CD33-expressing target cells by endowing more NK cells with killing function as compared to wild type HuM195 (19% with DLE HuM195 vs 1% with HuM195), by increasing their serial killing ability (5-19% involved in serial killing with DLE HuM195 vs 0-1% without antibody) and by stimulating their IFN-γ secretion. Dynamic timelapse monitoring showed that ADCC speeded up the kinetics of target conjugation, which was further increased by DLE HuM195. Secondly, DLE HuM195 necessitated fewer contacts to induce target apoptosis but also increased activation-induced cell death of NK cells. However, this increased AICD was independent of serial killing. Antibody-based therapies targeting tumor antigens will benefit from better understanding of cell-mediated tumor elimination. Single-cell analysis shows that Fc-modified mAb can significantly enhance NK cell killing of targets through improved recruitment of NK cells, conjugation kinetics, and serial killing. Enhanced target killing is also associated with increased AICD of NK cells, but the balance of effect favors the Fc modification for enhanced efficacy.

